# HIV, Tuberculosis, and Malaria Antimicrobial Resistance^1^

**DOI:** 10.3201/eid1011.040797_07

**Published:** 2004-11

**Authors:** J. Todd Weber, Gail Cassell

**Affiliations:** * Centers for Disease Control and Prevention, Atlanta, Georgia, USA;; † Eli Lilly and Company, Indianapolis, Indiana, USA

**Keywords:** HIV, Tuberculosis, Malaria, Antimicrobial Resistance

Increasing drug resistance among many infections of public health importance has created an urgent need for new and effective antimicrobial agents. However, existing economic incentives for research and development appear to be insufficient to meet the anticipated need for new antimicrobial drugs. U.S. Food and Drug Administration approval of new antibacterial agents has decreased by 56% in the past 20 years (1998–2002 vs. 1983–1987), and only 6 of 506 drugs disclosed in the developmental programs of the largest pharmaceutical and biotechnology companies are new antibacterial agents (*1*). Infectious diseases of worldwide importance such as tuberculosis and malaria are affected by the same problems, and antiretroviral drugs are not used effectively in developing nations because of the lack of economic incentives.

The Global Alliance for TB Drug Development is a public-private partnership; its goal is to help develop new, faster acting, and affordable tuberculosis medicines. The partnership intends to accelerate research and development and ensure affordability of the drugs developed, given that the need is greatest in resource-poor countries with high rates of tuberculosis. The Green Light committee reviews applications to the World Health Organization and its international partners for directly observed therapy short course (DOTS)-plus pilot projects in low- and middle-income countries, projects that modify typical DOTS strategy in light of issues (such as the use of second-line drugs) specific to areas with a high prevalence of multidrug-resistant tuberculosis.

The Medicines for Malaria Venture manages a portfolio of projects that are conducted by academic and pharmaceutical partners. Criteria for products to treat uncomplicated malaria include efficacy against drug-resistant strains, cure within 3 days, low propensity to generate rapid resistance, safety in children <6 years old and pregnant women, appropriate formulations and packaging, and low cost.

The Accelerating Access Initiative, a joint project of UNAIDS, other international organizations, and several HIV-drug–producing pharmaceutical companies, has developed what it calls a common approach to accelerating access to HIV/AIDS care and treatment in developing countries. The approach calls for political will and commitment of governments; strengthened national healthcare capacity; safe, secure, and efficient distribution systems; involvement of all sectors of society; substantial additional funding from national and international sources; and continued investment in research and development by the pharmaceutical industry.

## References

[1] Spellberg B, Power J, Brass EP, Miller LG, Edwards JE Jr. Trends in antimicrobial drug development: implications for the future. Clin Infect Dis. 2004;38:1279–86. 10.1086/420937 15127341

